# Altered Plasma Endocannabinoids and Oxylipins in Adolescents with Major Depressive Disorders: A Case–Control Study

**DOI:** 10.3390/nu18020280

**Published:** 2026-01-15

**Authors:** Akash Chakravarty, Abinaya Sreetharan, Ester Osuna, Isabelle Herter-Aeberli, Isabelle Häberling, Jeannine Baumgartner, Gregor E. Berger, Martin Hersberger

**Affiliations:** 1 Division of Clinical Chemistry and Biochemistry, Children’s Research Centre, University Children’s Hospital Zurich, University of Zurich, 8008 Zurich, Switzerland; 2 Laboratory of Human Nutrition, Institute of Food, Nutrition and Health, ETH Zurich, 8092 Zurich, Switzerland; 3 Laboratory of Nutrition and Metabolic Epigenetics, Institute of Food, Nutrition, and Health, ETH Zurich, 8092 Zurich, Switzerland; 4 Department of Child and Adolescent Psychiatry and Psychotherapy, Psychiatric Hospital, University of Zurich, 8008 Zurich, Switzerland; 5 Department of Nutritional Sciences, King’s College London, London SE1 9NH, UK; jeannine.baumgartner@kcl.ac.uk

**Keywords:** pMDD, endocannabinoids, oxylipins, PUFAs, inflammation

## Abstract

**Background:** Pediatric Major Depressive Disorder (pMDD) is one of the leading causes of disability in adolescents. There is currently no single explanation that fully accounts for the cause of the disorder, but various factors, including dysregulation of the immune and stress responses, have been linked to its onset. Oxylipins and endocannabinoids, derived from metabolization of *n*-3 and *n*-6 polyunsaturated fatty acids (PUFAs), regulate inflammation and have been suggested to attenuate inflammation associated with depression. This study aims to understand whether adolescents with pMDD have altered baseline levels of oxylipins and endocannabinoids compared to healthy adolescents. **Methods:** In this case–control study, we measured 60 oxylipins and endocannabinoids in plasma from 82 adolescents with pMDD and their matching healthy controls. **Results:** A Principal Component Analysis revealed substantial variability within each group and only a moderate degree of separation between them. In a paired analysis, the lipid mediators of controls exhibited higher concentrations of *n*-6 PUFA-derived prostaglandins and thromboxanes (PGE2, PGD2, PGF2a and TXB2), *n*-3 PUFA-derived TxB3, and the endocannabinoids AEA, EPEA, and DHEA. In contrast, cases had higher concentrations of the *n*-6 PUFA-derived 6-keto-PGF1a and the *n*-3 PUFA-derived PGD3. In addition, we observed a higher percentage of oxylipins and endocannabinoids derived from DHA (5.65 ± 5.46% vs. 4.72 ± 4.94%) and AA (16.31 ± 11.10% vs. 12.76 ± 13.46%) in plasma from controls, in line with the higher DHA and AA levels observed in erythrocytes from controls compared to cases. **Conclusions:** Overall, our results show lower plasma levels of endocannabinoids and lower DHA- and AA-derived oxylipins in adolescents with pMDD, supporting their role in the pathophysiology of pMDD. To infer a causative role of the *n*-3 and *n*-6 PUFA-derived oxylipins and endocannabinoids in pMDD, an intervention study with *n*-3 PUFA supplementation and monitoring of oxylipins and endocannabinoids would be necessary.

## 1. Introduction

Depression is recognized as a major cause of disability and premature death worldwide [1]. In adolescents, i.e., individuals between 10 and 19 years old, depression is a leading cause of illness and disability with a 12-month prevalence rate of 7.5% and a major risk factor for suicide, the second leading cause of death in this age group [2]. Worldwide epidemiological data suggest adolescent depression to be a global concern, with over one in five youth experiencing depressive episodes [3]. In a recent post-pandemic study in Switzerland, one-thirdof the recruited adolescent population screened positive for depression [4]. The disease often goes unnoticed, including by professionals, and the choice of treatment in pMDD is a major concern [5]. The current first-line treatments for pMDD are selective serotonin reuptake inhibitors (SSRIs) such as sertraline, citalopram, and paroxetine. However, they are not very effective and have been associated with several side effects [6].

In the last two decades, there have been several studies that have proposed the use of *n*-3 long-chain polyunsaturated fatty acids (LC-PUFAs), mainly eicosapentaenoic acid (EPA) and docosahexaenoic acid (DHA), as a potential therapy for depression [7,8]. Both pre-clinical and clinical studies have shown that these *n*-3 LC-PUFAs mitigate stress-related changes linked to depression, such as alteration of the monoaminergic neurotransmitter systems involving serotonin and dopamine [9]. In animal models, deprivation of *n*-3 PUFAs led to increased serotonin-2 and decreased dopamine-2 receptor density [10]. Clinical studies with *n*-3 LC-PUFA intervention, however, showed controversial results. A dose–response meta-analysis of randomized controlled trials found that 1 g/day of *n*-3 PUFA supplementation was associated with significant improvements in depressive symptoms in adults, with the greatest benefits seen at 1.5 g/day of combined EPA and DHA [11]. In adolescents, very few studies have reported the dosage regimen. A recent study reported using a dose range from 1.3 to 3.6 g/day of EPA and DHA; however, no clear efficacy of *n*-3 PUFA supplementation was observed [12]. A recent meta-analysis comprising five randomized clinical trials (RCTs) of *n*-3 LC-PUFA supplementation in adolescent depression suggests a minor reduction in self-reported depression symptoms, although with a very low certainty of evidence [8].

Mechanistically, inflammation plays a role in the pathophysiology of depression [13], and *n*-3 PUFAs possess immunomodulatory, anti-inflammatory, and pro-resolving properties [14,15]. They incorporate into cell membrane phospholipids, disrupt membrane rafts, and, when released from the membrane, suppress inflammatory signaling by activating PPAR-γ and free fatty acid receptor 4 [16,17]. PPAR-γ forms heterodimers with retinoid X receptor and represses pro-inflammatory transcription factors such as NF-κB upon activation, thereby reducing cytokine expression [18]. The free fatty acid receptor 4 (FFAR4, formerly known as GPR120) inhibits pro-inflammatory kinase cascades through β-arrestin-2 signaling and attenuates macrophage and microglial inflammatory responses [19]. In addition, *n*-3 PUFAs are metabolized into oxylipins, which are bioactive lipids produced enzymatically through the cyclooxygenase (COX), lipoxygenase (LOX), and the cytochrome P 450 (CYP) pathways [20]. The same enzymes also metabolize *n*-6 PUFAs into oxylipins, but the role of *n*-3 and *n*-6 oxylipins in inflammation differs substantially. Oxylipins derived from *n*-6 polyunsaturated fatty acids (PUFAs), such as prostaglandins and leukotrienes, primarily initiate and amplify inflammatory responses and are generally considered pro-inflammatory. In contrast, oxylipins generated from *n*-3 PUFAs, including resolvins, maresins, and protectins, play a central role in the resolution of inflammation by exerting potent anti-inflammatory and pro-resolution effects. [21,22].

In addition, the *n*-3 and *n*-6 PUFAs are metabolized to endocannabinoids, a class of bioactive lipids produced from *n*-3 and *n*-6 PUFAs by the action of *N*-acyltransferases and phospholipases, which serve as endogenous ligands for the cannabinoid receptors (CB1 and CB2) forming the endocannabinoid system [23]. These endocannabinoids are recognized to play a crucial role in the pathogenesis of depressive disorders and were shown to reduce inflammation [24]. The cannabinoid receptors CB1 and CB2 are G-protein-coupled receptors widely distributed in the central nervous system and peripheral tissues [25,26,27,28]. CB1 receptors are highly expressed in neuronal circuits involved in mood, cognition, and stress responses, and their activation modulates neurotransmitter release and synaptic plasticity [27], whereas CB2 receptors are predominantly found on immune cells and glia and regulate neuroimmune and inflammatory signaling [28]. The biosynthesis, signaling, and degradation of endocannabinoids are tightly regulated by specific enzymes, including fatty acid amide hydrolase (FAAH) and monoacylglycerol lipase (MAGL), which terminate endocannabinoid action after receptor engagement [26]. The endocannabinoid signaling network has been implicated in the modulation of stress responses, neuroinflammation, and emotional behavior, and dysregulation of the endocannabinoid system has been associated with the pathophysiology of depressive disorders [25,27,28].

To our knowledge, there is no study investigating plasma oxylipins and endocannabinoids in adolescents suffering from pMDD. To investigate whether the balance of pro-inflammatory to anti-inflammatory and pro-resolution oxylipins and endocannabinoids is altered in pMDD, we measured a panel of oxylipins and endocannabinoids derived from *n*-3 and *n*-6 PUFAs in the plasma of patients with pMDD and compared them to matched healthy controls (Figure S1).

## 2. Materials and Methods

### 2.1. Study Design

This is a single-center observational case–control study, which is an add-on study to the investigator-initiated clinical trial (IICT)—Omega-3 Fatty Acids as treatment for Paediatric Major Depressive Disorder Trial (Omega-3 pMDD) (SNSF 33IC30_166826 2016-02116). Cross-sectional data assessment was carried out in 82 13–18-year-old adolescents diagnosed with pMDD, recruited for the IICT, and 82 matching healthy controls. The matching of the healthy controls was performed in a 1:1 ratio according to demographic data, i.e., sex, age group, and educational level. Sample size calculation was performed as previously described [29]. Briefly, the sample size estimation was conducted using G*Power version 3.1.9.2 based on a logistic regression model with depression as a binary outcome, assessed using the Children’s Depression Rating Scale—Revised (CDRS-R). The model included ten co-variates and assumed a residual variance of R^2^ = 0.20. Under the assumption that a one-standard-deviation increase in the continuous predictor corresponded to odds ratios between 1.5 and 2.0, the analysis indicated that a total sample of approximately 200 participants with a 1:1 case–control ratio would provide more than 80% power to detect medium to large effect sizes at a two-sided significance level of 0.05. Although this target sample size was initially planned and was used for other studies with the same cohort [19], the final analytical sample size for this current study comprised 82 cases and 82 controls due to loss of samples during collection and storage.

The recruitment of the cases, i.e., adolescents diagnosed with pMDD, was performed as described previously [30] in the period between May 2017 and June 2021. The cases recruited for this study were male and female participants, randomly selected from the Omega-3 pMDD study cohort to match the controls.

For the control participants, the recruitment was performed through schools, social media, and leisure time clubs from the Canton of Zurich and the surrounding German-speaking Cantons of Switzerland at the Laboratory of Human Nutrition at ETH Zurich, Switzerland, from September 2019 until December 2020.

Recruitment of cases and controls was performed in two age groups: the first age group comprising participants between 13 and<16 years of age, and the second age group comprising participants between 16 and <18 years of age. All adolescents in the younger age group attended lower secondary school level. However, the adolescents in the older age group were further matched based on their higher secondary school educational level (*n* = 26 from each level): (1) vocational education (apprenticeship) and (2) baccalaureate/vocational baccalaureate.

The inclusion and exclusion criteria for the cases and controls have been described previously [29]. Briefly, the cases comprised in- or outpatients with a current primary diagnosis of Major Depressive Disorder (single or recurrent) according to DSM-IV criteria [31], having a CDRS-R total score of ≥40 [32], confirmed using the K-SADS-PL, and with at least moderate symptom severity. Participants were required to be medically stable, able to comply with study procedures, and free of clinically significant laboratory abnormalities. Biological samples were collected at baseline before randomization of the cases. Controls were age- and sex-matched participants without a current psychiatric diagnosis, as assessed by M.I.N.I. KID, and without chronic medication use. For both groups, exclusion criteria included recent regular omega-3 supplementation, pregnancy, or breastfeeding, relevant neurological or medical conditions, substance dependence, major psychiatric comorbidities, or inability to comply with study procedures.

The study was approved by the ethics committee of the Canton of Zurich (BASEC-Nr. 2019-00717) and registered at www.clinicaltrials.gov (NCT04158869). The study was approved as an add-on study to the investigator-initiated clinical trial (SNSF 33IC30_166826, BASEC-Nr. 2016-02116). All caregivers and adolescents ≥14 years of age gave their written informed consent, and adolescents <14 years of age gave their oral assent before any research-related assessments were conducted.

### 2.2. Sample Collection and Pre-Analytics

Blood samples for the cases and controls were collected by study nurses. Blood was collected into EDTA-coated tubes (for controls: BD Vacutainer; for cases: Sarstedt), and plasma was extracted using centrifugation at 2113× *g* for 10 min at 4 °C within 15 min of collection. The extracted plasma was immediately stored in Eppendorf tubes at −80 °C until the samples were subjected to oxylipin and endocannabinoid analysis.

### 2.3. Measurement of Oxylipins and Endocannabinoids in Plasma

The plasma samples were thawed for 20 min at room temperature. A total of 200 µL plasma was diluted 1:1 with methanol, and an internal standard mixture for the oxylipins and endocannabinoids was added to each sample in equal quantities (25 µL), as described previously [33]. The diluted samples were vortexed for 10 s and then centrifuged at 10,000× *g* for 5 min at 4 °C. The supernatants were collected after centrifugation in a 96-well collection plate and further diluted with 1.4 mL of Milli-Q water. The samples were then subjected to solid-phase extraction (SPE) using 96-well reverse-phase SPE plates (Strata-X, Phenomenex, Torrance, CA, USA) on a 96-well positive-pressure SPE manifold (Biotage, Uppsala, Sweden). After the SPE, the samples were eluted with methanol and evaporated on a 96-well evaporation device (Biotage, Uppsala, Sweden). The samples were then reconstituted in 50 µL of methanol and transferred to HPLC vials for UHPLC-MS/MS analysis.

### 2.4. Instrumental Setup

The UHPLC-MS/MS analysis was carried out using a Shimadzu Nexera X2 UHPLC system, comprising an SIL-30AC autosampler, SIL-20AC column oven, and LC-30AD pumps (Shimadzu Schweiz GmbH, Reinach, Switzerland) coupled to a Sciex 6500+ triple quadrupole mass spectrometer (AB Sciex, Zug, Switzerland) [33]. Oxylipins and endocannabinoids were separated on a Waters Acquity UPLC CSH C18 Van Guard Premier column (1.7 µm, 2.1 × 150 mm; Waters AG, Baden-Dättwil, Switzerland), maintained at 40 °C. Separation was achieved using a gradient elution with mobile phase A consisting of Milli-Q water with 0.28% ammonium hydroxide, and mobile phase B comprising a methanol/acetonitrile mixture (70:30). The gradient program was as follows: 25% B at 0 min, 35% at 0.5 min, 60% at 10 min, 90% at 10.5 min, and 90% at 15 min. The column was then re-equilibrated to initial conditions for 5 min before the next sample run. A sandwich injection technique was used to accommodate samples dissolved in pure methanol, in which the autosampler introduced 10 µL of sample flanked by 1 µL of air and 15 µL of mobile phase A before, and 1 µL of air with 10 µL of mobile phase A after the sample.

The Sciex 6500+ operated in polarity-switching mode to detect both positive and negative ions. Source parameters for negative-ion ion detection included curtain gas at 35, collisionally activated dissociation (CAD) gas at 9, ion source gases 1 and 2 at 70, ion spray voltage at −4500 V, and source temperature at 500 °C. Oxylipins and endocannabinoids were identified using multiple reaction monitoring (MRM) based on one precursor ion and two product ions per compound. Collision energy values were individually optimized for each ion transition. Declustering potential (DP), entrance potential (EP), and collision cell exit potential (CXP) were consistently set at −40, −10, and −15 V, respectively. For positive-ion ion detection, the same settings were used but with corresponding positive voltages. The scan duration for each MRM transition was 0.8 s, and a 90 s retention time window was applied.

### 2.5. Data Analysis

Mass spectrometric data for the panel of 60 oxylipins and endocannabinoids and their corresponding internal standards were analyzed using SciexOS^®^ (version 3.0.0). All statistical data analyses were performed using R Studio v4.4.3 (R foundation for statistical computing, Vienna, Austria) and Graphpad Prism 9 (GraphPad Software, La Jolla, CA, USA). For samples where the concentration of measured oxylipins was lower than the previously determined LLOQ, the values were replaced by ½ of the LLOQ for the specific oxylipin according to Hartling et al. [31]. A S/N ratio threshold of 5 was used for data analysis. The recently published technical recommendations for quantification of oxylipins using LC-MS were taken into account, and the nomenclature of the oxylipins in our panel was determined accordingly (Table S1) [32]. Principal Component Analysis (PCA) was used to study the difference in the overall targeted plasma lipidome between cases and matched controls. Prior to PCA, oxylipin and endocannabinoid concentrations were log-transformed and converted to z-scores to ensure that all variables contributed equally to the analysis regardless of scale. PCA was conducted using the prcomp function in R, which is based on singular-value decomposition of the centered and scaled data matrix. The analysis was performed on the transposed data matrix, such that samples were treated as observations and concentrations of oxylipins and endocannabinoids as variables. Principal components were extracted without rotation, and the proportion of variance explained by each component was assessed using eigenvalues. Group separation was visualized in score plots, with 95% confidence ellipses computed for each group. Variable loadings were visualized and color-coded according to PUFA class. PCA results were used for exploratory purposes only, and no assumptions of group separation or normality were imposed. A paired Wilcoxon signed-rank test was used to assess individual quantitative differences in oxylipin and endocannabinoid levels between cases and controls. The data are presented as volcano plots, with *p*-values derived from the Wilcoxon signed-rank test. The statistical differences in the percentage of oxylipins and endocannabinoids derived from all the fatty acids and the total concentration of oxylipins and endocannabinoids between cases and controls were calculated using the Wilcoxon rank-sum test. For all statistical analyses, a *p*-value < 0.05 was considered significant.

## 3. Results

### 3.1. Differences in the Lipidome Between Cases and Controls

**Figure 1:**
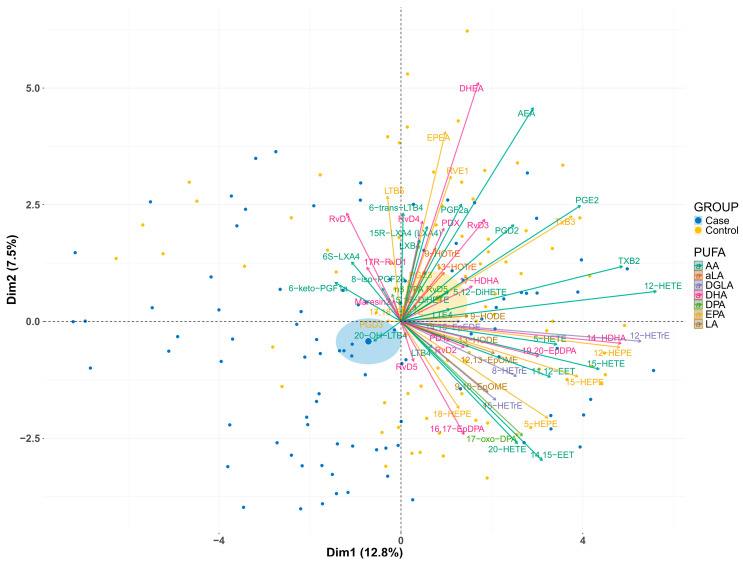
PCA plot illustrating the lipidome of adolescents with pMDD (*n* = 82) and their matching healthy controls (*n* = 82). Oxylipin and endocannabinoid concentrations were log2-transformed and converted to z-scores. Every dot in the graphical illustration represents the combined positioning of 60 oxylipins and endocannabinoids measured in adolescents with pMDD (blue) and in controls (yellow). The contribution of each oxylipin and endocannabinoid to the separation of the two groups is displayed as a vector in the color of the parental PUFA.

Principal Component Analysis (PCA) was performed to observe the differences in the overall lipidome between the 82 cases and their matching healthy controls (Figure 1). The results show moderate differences between the lipidome of cases (blue) and controls (yellow) (PC1: 12.8%, PC2: 7.5%) and substantial variability within the groups. In general, we observe that the vectors for the majority of oxylipins point towards the control group.

**Figure 2:**
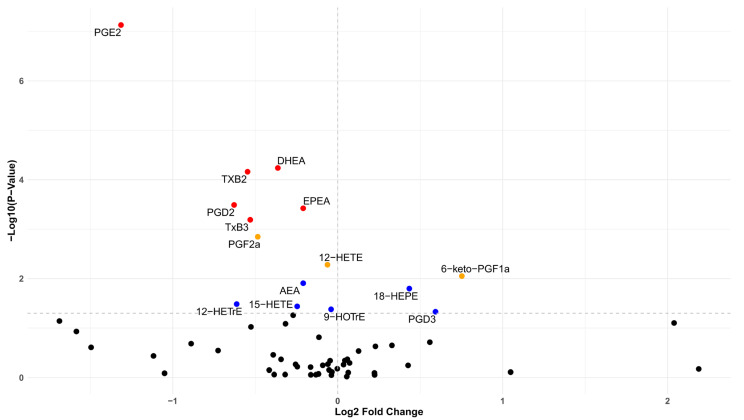
Volcano plot of two-tailed paired *t*-tests comparing the concentration of oxylipins and endocannabinoids in plasma of cases (right) to controls (left) with significance levels of *p* < 0.05 (horizontal dotted line). Highly significant: *p* < 0.001 (Red); significant: *p* < 0.01 (Yellow); marginally significant: *p* < 0.05 (Blue); not significant: *p* > 0.05 (Black).

To identify which oxylipins mainly contribute to the separation of the two groups, we performed a volcano plot analysis between cases and controls. In the volcano plot, we observed that AA- and EPA-derived prostaglandins and thromboxanes produced by COX enzymatic activity (PGE2, PGD2, PGF2a, TXB2, and TXB3) and ALA-, AA-, and DGLA-derived 9-HOTrE, 12-HETrE, 12-HETE, and 15-HETE produced by the enzymatic activity of ALOX5, ALOX12 and ALOX15 are higher in controls than in cases with pMDD (*p* < 0.01) (Figure 2). In addition, we observed higher levels of the endocannabinoids AEA (from AA), DHEA (from DHA), and EPEA (from EPA) produced by *N*-acyltransferases in controls [33].

In contrast, only the AA-derived COX metabolite 6-keto-PGF1a, the EPA-derived CYP-hydroxylation product 18-HEPE, and the EPA-derived COX metabolite PGD3 showed higher levels in cases than in controls (Figure 2).

**Figure 3:**
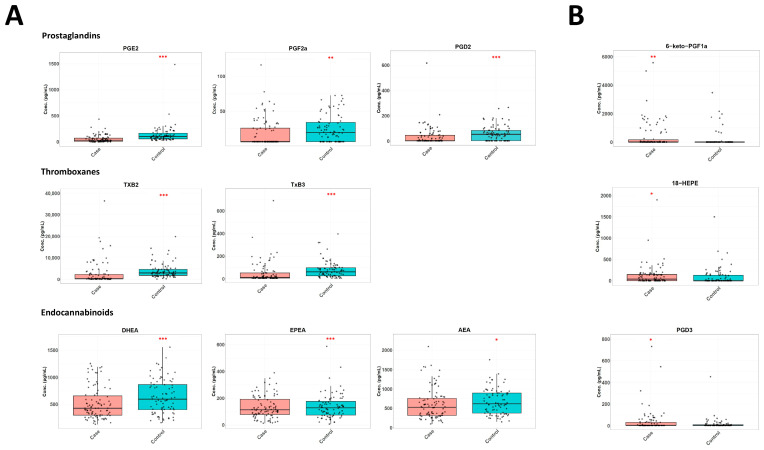
Box plots showing the distribution in concentration of oxylipins and endocannabinoids: (**A**) higher in controls compared to cases; (**B**) higher in cases compared to controls. (*n* = 82 each, for cases and controls.) Every dot represents an individual participant. Wilcoxon signed-rank test. *p* < 0.001 = ***, *p* < 0.01 = **, *p* < 0.05 = *. Horizontal bold line in box plots represents the median, the box margins represent the 25th and 75th percentile, and the vertical bold line represents the 5th and 95th percentile.

To investigate the variability of the oxylipin and endocannabinoid concentrations within the groups and between controls and cases, we displayed the levels of the oxylipins and endocannabinoids, which showed significant differences in concentration between controls and cases as box plots (Figure 3 and Figure S1). The box plots for all oxylipins and endocannabinoids showed high variability within groups and a large overlap between groups (Figure 3 and Figure S1).

### 3.2. Contribution of Individual PUFAs to Lipidomic Differences

**Figure 4:**
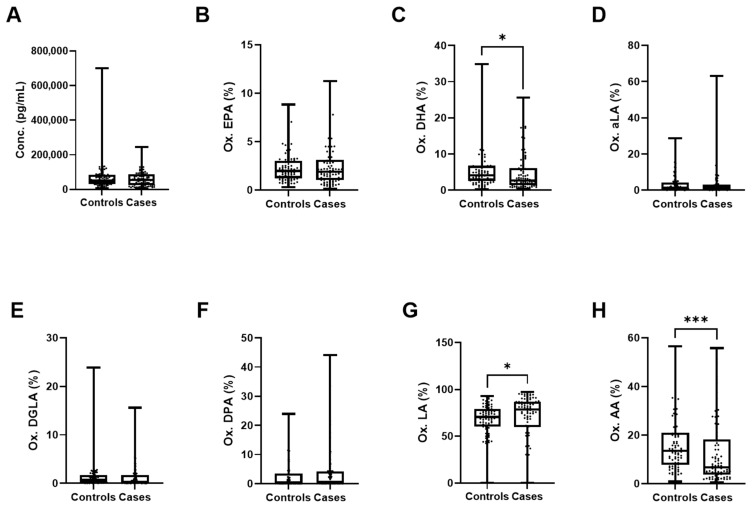
Percentage of oxylipins and endocannabinoids according to parental PUFAs they are derived from controls and cases. (**A**) Total concentration of all oxylipins and endocannabinoids in our panel. Percentage of oxylipins and endocannabinoids derived from (**B**) EPA, (**C**) DHA, (**D**) aLA, (**E**) DGLA, (**F**) DPA, (**G**) LA, and (**H**) AA. Wilcoxon rank-sum test. *p* < 0.001 = ***, *p* < 0.05 = *. Ox., oxylipins.

Since the qualitative data of the PCA plot (Figure 1) showed a majority of the oxylipin and endocannabinoid vectors pointing towards the control group, we compared the overall oxylipin and endocannabinoid concentrations between the two groups. No significant differences were found in total oxylipin and endocannabinoid concentrations between controls and cases (68,715 ± 78,455 vs. 61,680 ± 46,559 pg/mL) (Figure 4A), but again, a large variability was observed within groups. In a previous analysis of this case–control cohort, we observed higher proportions of the *n*-3 PUFAs EPA, DPA, and DHA, as well as the *n*-6 PUFAs DGLA and AA, in erythrocyte phospholipids from controls compared to individuals with pMDD [29]. Based on these findings, we subsequently compared the percentage of oxylipins derived from each PUFA between controls and cases (Figure 4). In accordance with the PUFA results in phospholipids of erythrocytes, significantly higher percentages of oxylipins and endocannabinoids derived from DHA (5.65 ± 5.46% vs. 4.72 ± 4.94%, *p* = 0.020) (Figure 4C) and AA (16.31 ± 11.10% vs. 12.76 ± 13.46%, *p* = 0.0005) (Figure 4H) were observed in controls. In contrast, a higher percentage of oxylipins from LA (67.91 ± 17.43% vs. 71.53 ± 22.26%, *p* = 0.014) was observed in cases (Figure 4G).

## 4. Discussion

In our case–control study, we investigated whether the balance of pro-inflammatory, anti-inflammatory, and pro-resolution oxylipins and endocannabinoids differs between adolescents with pMDD and matched healthy controls. Although the overall targeted plasma lipidome profiles showed only moderate separation and substantial within-group variability, we identified small but significant differences in specific oxylipins and endocannabinoids. Controls exhibited higher concentrations of AA- and EPA-derived prostaglandins and thromboxanes (PGE2, PGD2, PGF2a, TXB2, and TXB3) of aLA-, AA-, and DGLA-derived 9S-HOTrE, 12S-HETrE, 12-HETE, and 15S-HETE, and of the endocannabinoids AEA, DHEA, and EPEA, while the AA-derived 6-keto-PGF1a and the EPA-derived 18-HEPE and PGD3 were higher in cases than in controls.

From these mainly minor differences between adolescents with pMDD and controls, the difference in the endocannabinoid levels stands out because all three endocannabinoids investigated were lower in plasma from adolescents with pMDD than from controls (Table S2). These findings are in line with a recent study on the same participant cohort that showed reduced AEA levels in hair of adolescents with pMDD compared to controls [34], while previous studies investigating associations of plasma levels of endocannabinoids with MDD in adults showed conflicting results [24]. Most studies investigating associations between AEA levels and MDD in adults showed no significant differences in AEA levels between patients and controls [35,36,37,38,39,40]. Similarly, one study investigating peripheral levels of DHEA showed no association of DHEA levels with MDD and no correlation of DHEA levels with the severity of depression [37]. In contrast, the only study measuring EPEA levels in an intervention trial with EPA and DHA showed a positive correlation between EPEA levels and clinical remission rates in MDD [41]. In this line, there is pharmacological and molecular evidence for a role of these *N*-acylethanolamines in depression.

Several mechanisms leading to an anti-depressive effect have been discussed for the endocannabinoids, including modulation of serotonergic neurotransmission in the prefrontal cortex and hippocampus, modulation of noradrenergic neurotransmission, regulation of neurotrophic factors, tonic inhibition of the hypothalamic–pituitary–adrenal axis, and an anti-inflammatory effect [42,43]. These effects are mediated by a set of receptors that show differential expression between the central nervous system and the periphery and are differentially triggered by the endocannabinoids [44]. While the *n*-6-derived *N*-acylethanolamines like AEA were shown to signal through CB1, CB2, and the TRPV1 receptors, the *n*-3-derived EPEA and DHEA seem to mainly trigger CB2 receptors [45]. Signaling through the CB1 receptors mainly impacts neurotransmitter regulation and stress response in the central nervous system, while signaling through the mainly peripheral CB2 receptors reduces inflammation [42,46,47]. In our study, adolescents with pMDD have lower levels of AEA, EPEA, and DHEA than controls, suggesting a lack of beneficial effects on neurotransmitter regulation, stress response, and inflammation. These results are in line with the reduction in levels of conventional inflammatory markers such as *C*-reactive protein (CRP) and alpha-1-acid-glycoprotein (AGP) in controls in comparison to the cases, which were studied for the same cohort as our current study in another recent study [48], indicating a less inflammatory signature in the healthy controls. However, we have to be cautious with the interpretation of our results, because we only measured three out of hundreds of *N*-acylethanolamines and did not measure the ten-times-higher-concentrated2-acylglycerol endocannabinoids, like 2-arachidoylglycerol or the lipoamino acids [44,49,50]. In addition, there is an indication that the endocannabinoids are further metabolized to endocannabinoid epoxides by cyclooxygenases (COXs), lipoxygenase (LOX), and CYP-450 epoxygenases, and that these endocannabinoid epoxides have anti-inflammatory effects also [51,52,53]. Nevertheless, the observed reduction in three *N*-acylethanolamines in adolescents with pMDD compared to healthy controls is in line with the anti-depressive effect of these endocannabinoids [42].

Our finding also support that the endocannabinoid and the oxylipin profiles are modified by tissue PUFA levels [54,55,56,57] because we observe a general decrease in *N*-acylethanolamines derived from both the *n*-6 and *n*-3 PUFA families in adolescents with pMDD, which are in line with the reduced EPA, DHA, and AA levels in erythrocytes of adolescents with pMDD in our case–control study [29]. However, this was not the case for oxylipins from all PUFAs because the percentage of LA oxylipins is higher in adolescents with pMDD, although the percentage of LA in phospholipids of erythrocytes did not differ between cases and controls [29]. The LA-derived oxylipins contributing most to this difference are 9- and 13-HODE, two oxylipins associated with oxidative stress [58]. However, we did not observe an increase in markers for oxidative stress in adolescents with pMDD because the concentrations of these two oxylipins and of 8−iso−PGF2a, another marker for oxidative stress [59], are not different between controls and adolescents with pMDD. This indicates that the percent increase for 9- and 13-HODE in adolescents with pMDD may be due to the reduction in the percentage of endocannabinoids and other oxylipins.

Another finding in our case–control study is that plasma levels of the *n*-6 PUFA-derived prostaglandins (PGE2, PGD2, and PGF2a) and thromboxanes (TxB2) are higher in controls than in cases. This contradicts studies from the early 1980s that which showed increased levels of these prostaglandins in patients with MDD [60,61] but is in line with recent mouse studies that showed an inverse association of PGD2 levels with depression [62]. This discrepancy may arise from pre-analytical differences because factors like tube composition and the delay between blood draw and centrifugation are known to affect measured prostaglandin and thromboxane levels in plasma [31,63]. In our case–control study, we used EDTA tubes from two different suppliers for the collection of blood from adolescents with pMDD and controls, and we collected blood at wards of different hospitals from adolescents with pMDD, while another specialized team collected blood from healthy controls. We, therefore, cannot exclude that pre-analytical differences between samples from adolescents with pMDD and controls may have led to the observed higher prostaglandin and thromboxane levels in controls.

Similarly, we do not want to overemphasize the observed differences in the AA-derived 6-keto-PGF-1a, and the EPA-derived 18-HEPE and PGD3 between adolescents with pMDD and controls because these oxylipins were only measurable in a minority of cases and controls. Most measurements resulted in concentrations below the LLOQ for our UHPLC-MS/MS method [31], which may have led to an overestimation of the differences between groups because, for statistical analysis, values below the LLOQ were imputed as half the LLOQ.

## 5. Conclusions

Overall, our results show only a modest association between endocannabinoids and oxylipins with depression in adolescents. The strength of this study is the large sample size in comparison to previous studies and the large number of oxylipins and endocannabinoids analyzed simultaneously. The main limitation of this study is the different teams and procedures used for sampling between cases with pMDD and the healthy controls. As mentioned in the Discussion, this could lead to differences in platelet activation and, thus, to differences in the production of COX- and ALOX12-derived prostaglandins, thromboxanes, and other oxylipins. Whether the observed differences in endocannabinoids and oxylipins contribute to disease development or are a consequence of the underlying pathophysiology remains to be determined in a randomized controlled intervention trial with *n*-3 fatty acids in adolescents with pMDD.

## Data Availability

The original contributions presented in this study are included in the article/Supplementary Materials. Further inquiries can be directed to the corresponding author.

## Supplementary Materials

The following supporting information can be downloaded at: https://www.mdpi.com/article/10.3390/nu18020280/s1, Table S1: Nomenclature of oxylipins and endocannabinoids according to the technical recommendations [32]. Figure S1: Box plots of plasma oxylipins and endocannabinoids in pg/mL in controls and cases; Table S2: Concentration (pg/mL) of oxylipins and endocannabinoids in controls and cases.

## Abbreviations

The following abbreviations are used in this manuscript:

PGE2	Prostaglandin E2
PGD2	Prostaglandin D2
PGF2a	Prostaglandin F2a
TXB2	Thromboxane B2
TxB3	Thromboxane B3
AEA	Arachidonoyl ethanolamide
EPEA	Eicosapentaenoyl ethanolamide
DHEA	Docosahexaenoyl ethanolamide
PGF1a	Prostaglandin F1a
PGD3	Prostaglandin D3
AA	Arachidonic acid
SSRIs	Selective serotonin reuptake inhibitors
LC-PUFAs	Long-chain polyunsaturated fatty acids
PPAR-γ	Peroxisome Proliferator-Activated Receptor gamma
FFAR4	Free fatty acid receptor 4
GPR120	G Protein-coupled receptor 120
FAAH	Fatty acid amide hydrolase
MAGL	Monoacylglycerol lipase
SNSF	Swiss National Science Foundation
DSM-IV	Diagnostic and Statistical Manual of Mental Disorders, Fourth Edition
CDRS-R	Children’s Depression Rating Scale-Revised
K-SADS.PL	Kiddie Schedule for Affective Disorders and Schizophrenia
M.I.N.I. KID	Mini-International Neuropsychiatric Interview for Children and Adolescents
EDTA	Ethylenediaminetetraacetic acid
HPLC	High-performance liquid chromatography
UHPLC-MS/MS	Ultra-high-performance liquid chromatography-tandem mass spectrometry
LLOQ	Lower limit of quantification
S/N	Signal-to-noise ratio
ALOX5	Arachidonate 5-lipoxygenase
DGLA	Dihomo-γ linoleic acid
LA	Linoleic acid
aLA	α-linolenic acid
DPA	Docosapentaenoic acid
HOTrE	Hydroxyoctadecatrienoic acid
HETrE	Hydroxyeicosatrienoic acid
HETE	Hydroxyeicosatetraenoic acid
HEPE	Hydroxyeicosapentaenoic acid
CB1	Cannabinoid receptor 1
CB2	Cannabinoid receptor 2
TRPV1	Transient receptor potential vanilloid 1
HODE	Hydroxyoctadecadienoic acid
