# Supplementary material for: Altered Plasma Endocannabinoids and Oxylipins in Adolescents with Major Depressive Disorders: A Case–Control Study
Source: Nutrients. 2026 Jan 15;18(2):280. doi: 10.3390/nu18020280 (PMC12844611; doi:10.3390/nu18020280)
Supplement: Supplementary file 1 [file nutrients-18-00280-s001.zip › nutrients-4042816-supplementary.pdf]

Supplementary Information

Table S1. Nomenclature of oxylipins and endocannabinoids according to the technical recommendations [1].

Abbreviations of oxylipins used in this research study	Chemical Name	Abbreviations of oxylipins as per the technical recommendations
6-keto-PGF1a	9α,11α,15S-trihydroxy-6-oxo-prost-13E-en-1-oic acid	6-keto-PGF1a
TxB3	9α,11,15S-trihydroxy-thromba-5Z,13E,17Z-trien-1-oic acid	TxB3
RVE1	5S,12R,18R-trihydroxy-6Z,8E,10E,14Z,16E-eicosapentaenoic acid	5,12,18-TriHEPE
PGE3	9-oxo-11α,15S-dihydroxy-prosta-5Z,13E-dien-1-oic acid	PGE3
PGD3	9α,15S-dihydroxy-11-oxo-prosta-5Z,13E,17Z-trien-1-oic acid	PGD3
20-OH-LTB4	5S,12R,20-trihydroxy-6Z,8E,10E,14Z-eicosatetraenoic acid	20-OH-LTB4
8-iso-PGF2a	9α,11α,15S-trihydroxy-(8β)-prosta-5Z,13E-dien-1-oic acid	8-iso-PGF2a
TXB2	9α,11,15S-trihydroxythromba-5Z,13E-dien-1-oic acid	TXB2
LTE4	5S-hydroxy-6R-(S-cysteinyl)-7E,9E,11Z,14Z-eicosatetraenoic acid	LTE4
PGE2	9-oxo-11α,15S-dihydroxy-prosta-5Z,13E-dien-1-oic acid	PGE2
PGD2	9α,15S-dihydroxy-11-oxo-prosta-5Z,13E-dien-1-oic acid	PGD2
PGF2a	9α,11α,15S-trihydroxy-prosta-5Z,13E-dien-1-oic acid	PGF2a
LXB4	5S,14R,15S-trihydroxy-6E,8Z,10E,12E-eicosatetraenoic acid	5,14,15-TriHETE
RvD2	7S,16R,17S-trihydroxy-4Z,8E,10Z,12E,14E,19Z-docosahexaenoic acid	7,16,17-TriHDHA
RvD3	4S,11R,17S-trihydroxy-5Z,7E,9E,13Z,15E,19Z-docosahexaenoic acid	4,11,17-TriHDHA
17R-RvD1	7S,8R,17R-trihydroxy-4Z,9E,11E,13Z,15E,19Z-docosahexaenoic acid	7(S),8(R),17(R)-TriHDHA
RvD1	7S,8R,17S-trihydroxy-4Z,9E,11E,13Z,15E,19Z-docosahexaenoic acid	7(S),8(R),17(S)-TriHDHA
15R-LXA4 (LXA4)	5,6,15-trihydroxy-7E,9E,11Z,13E-eicosatetraenoic acid	5, 6, 15-TriHETE
6S-LXA4	5S,6S,15S-trihydroxy-7E,9E,11Z,13E-eicosatetraenoic acid	5(S),6(S),15(S)-TriHETE
LTB5	5S,12R-dihydroxy-6Z,8E,10E,14Z,17Z-eicosapentaenoic acid	5,12-DiHEPE
RvD4	4S,5R,17S-trihydroxy-6E,8E,10Z,13Z,15E,19Z-docosahexaenoic acid	4,11,17-TriHDHA
6-trans-LTB4	5S,12R-dihydroxy-6E,8E,10E,14Z-eicosatetraenoic acid	5,12-DiHETE
PDX	10(S),17(S)-dihydroxy-4Z,7Z,11E,13Z,15E,19Z-docosahexaenoic acid	10(S),17(S)-DiHDHA
5,15-DiHETE	5S,15S-dihydroxy-6E,8Z,10Z,13E-eicosatetraenoic acid	5,15-DiHETE
RvD5	7S,17S-dihydroxy-4Z,8E,10Z,13Z,15E,19Z-docosahexaenoic acid	7,17-DiHDHA
Maresin1	7R,14S-dihydroxy-4Z,8E,10E,12Z,16Z,19Z-docosahexaenoic acid	7,14-DiHDHA
PD1	10R,17S-dihydroxy-4Z,7Z,11E,13E,15Z,19Z-docosahexaenoic acid	10(R),17(S)-TriHDHA
n3 DPA RvD5	7S,17S-dihydroxy-8E,10Z,13Z,15E,19Z-docosapentaenoic acid	7,17-DiHDPA
LTB4	5S,12R-dihydroxy-6Z,8E,10E,14Z-eicosatetraenoic acid	5(S),12(R)-DiHETE
9-HOTrE	9S-hydroxy-10E,12Z,15Z-octadecatrienoic acid	9-HOTrE
Maresin2	13R,14S-dihydroxy-4Z,7Z,9E,11E,16Z,19Z-docosahexaenoic acid	13,14-DiHDHA
5,12-DiHETE	5S,12S-dihydroxy-6E,8Z,10E,14Z-eicosatetraenoic acid	5(S),12(S)-DiHETE
18-HEPE	(±)-18-hydroxy-5Z,8Z,11Z,14Z,16E-eicosapentaenoic acid	18-HEPE
17,18-EpETE	(17S,18R)-epoxy-5Z,8Z,11Z,14Z-eicosatetraenoic acid	17,18-EpETE
15-HEPE	15-hydroxy-5Z,8Z,11Z,13E,17Z-eicosapentaenoic acid	15-HEPE
20-HETE	20-hydroxy-5Z,8Z,11Z,14Z-eicosatetraenoic acid	20-HETE
12-HEPE	(±)-12-hydroxy-5Z,8Z,10E,14Z,17Z-eicosapentaenoic acid	12-HEPE
13-HODE	(±)-13-hydroxy-9Z,11E-octadecadienoic acid	13-HODE
9-HODE	(±)-9-hydroxy-10E,12Z-octadecadienoic acid	9-HODE
5-HEPE	5S-hydroxy-6E,8Z,11Z,14Z,17Z-eicosapentaenoic acid	5-HEPE
12,13-EpOME	(12S,13R)-epoxy-9Z-octadecenoic acid	12,13-EpOME
15-HETE	15S-hydroxy-5Z,8Z,11Z,13E-eicosatetraenoic acid	15-HETE

9,10-EpOME	(9R,10S)-epoxy-12Z-octadecenoic acid	9,10-EpOME
14,15-EET	(14S,15R)-epoxy-5Z,8Z,11Z-eicosatrienoic acid	14,15-EET
17-HDHA	(±)17-hydroxy-4Z,7Z,10Z,13Z,15E,19Z-docosahexaenoic acid	17-HDHA
12-HETE	(±)12-hydroxy-5Z,8Z,10E,14Z-eicosatetraenoic acid	12-HETE
19,20-EpDPA	(19S,20R)-epoxy-4Z,7Z,10Z,13Z,16Z-docosapentaenoic acid	19,20-EpDPA
17-oxo-DPA	7Z,10Z,13Z,15E,19Z-17-oxo-docosapentaenoic acid	17-oxo-DPA
14-HDHA	(±)14-hydroxy-4Z,7Z,10Z,12E,16Z,19Z-docosahexaenoic acid	14-HDHA
11,12-EET	10-[(2R,3S)-3-(2Z)-2-octen-1-yl-2-oxiranyl]-5Z,8Z-decadienoic acid	11,12-EET
16,17-EpDPA	(16S,17R)-epoxy-4Z,7Z,10Z,13Z,19Z-docosapentaenoic acid	16,17-EpDPA
15-HETrE	15S-hydroxy-8Z,11Z,13E-eicosatrienoic acid	15-HETrE
14,15-EpEDE	13-[(2R,3S)-(3-pentyl-2-oxiranyl)]-8Z,11Z-tridecadienoic acid	14,15-EpEDE
12-HETrE	12S-hydroxy-8Z,10E,14Z-eicosatrienoic acid	12-HETrE
5-HETE	5S-hydroxy-6E,8Z,11Z,14Z-eicosatetraenoic acid	5-HETE
EPEA	N-(2-hydroxyethyl)-5Z,8Z,11Z,14Z,17Z-eicosapentaenamide	EPEA
DHEA	N-(2-hydroxyethyl)-4Z,7Z,10Z,13Z,16Z,19Z-docosahexaenamide	DHEA
AEA	N-(2-hydroxyethyl)-5Z,8Z,11Z,14Z-eicosatetraenamide	AEA
13-HOTrE	13S-hydroxy-9Z,11E,15Z-octadecatrienoic acid	13-HOTrE
8-HETrE	8S-hydroxy-9E,11Z,14Z-eicosatrienoic acid	8-HETrE

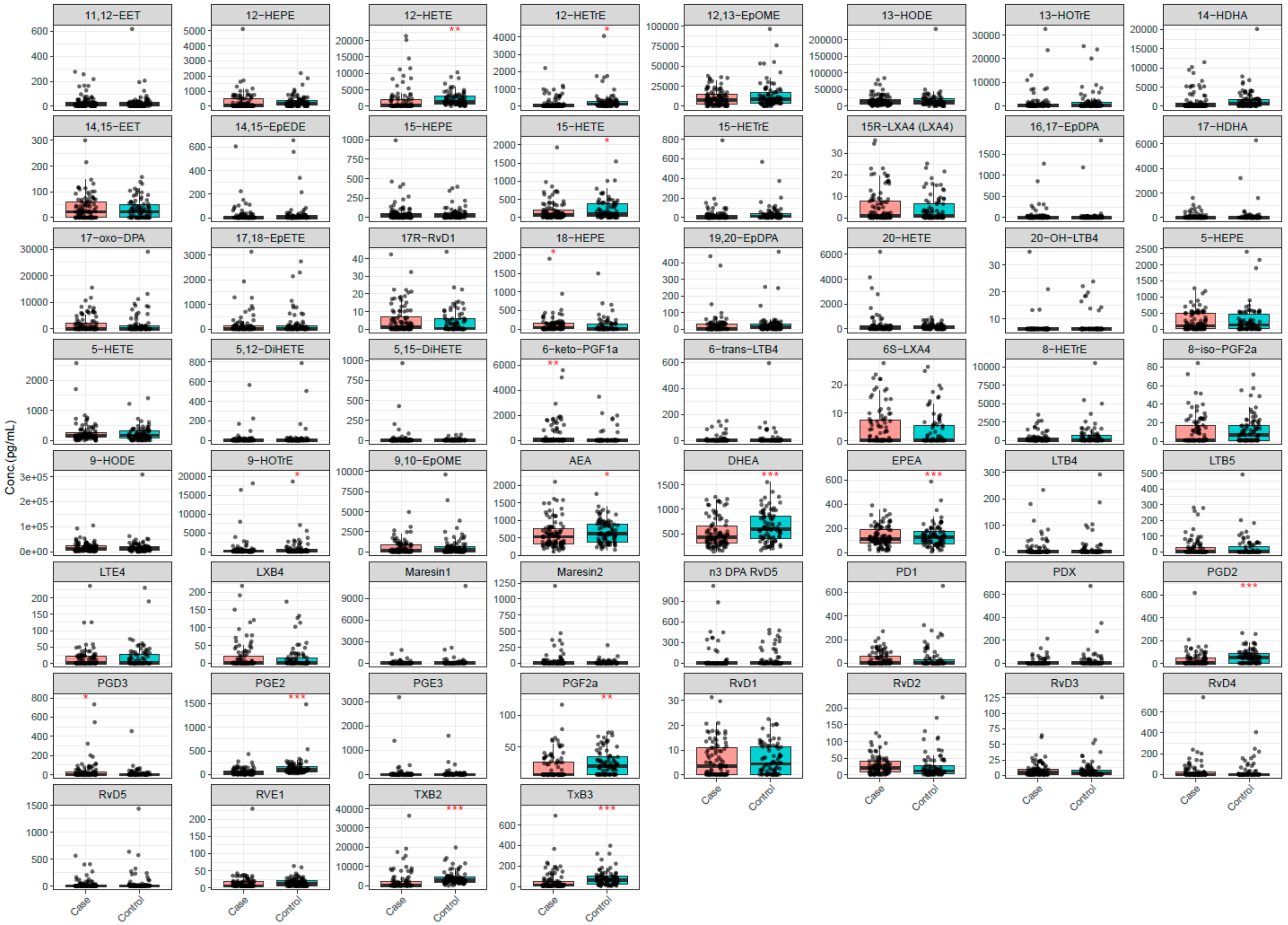

Figure S1. Box plots of plasma oxylipins and endocannabinoids in pg/mL in controls and cases.

Table S2. Concentration (pg/mL) of oxylipins and endocannabinoids in controls and cases.

Oxylipin	PUFA	Type	Controls (pg/mL)	Cases (pg/mL)
11,12-EET	AA	n-6	32.57 ± 74.46	33.57 ± 55.08
12-HETE	AA	n-6	2131.41 ± 2078.12	2041.37 ± 4219.11
14,15-EET	AA	n-6	32.30 ± 38.24	33.75 ± 45.21
15R-LXA4 (LXA4)	AA	n-6	4.32 ± 6.07	4.20 ± 6.13
15-HETE	AA	n-6	227.74 ± 275.82	192.05 ± 300.60
20-HETE	AA	n-6	185.15 ± 220.07	382.88 ± 947.24
20-OH-LTB4	AA	n-6	7.53 ± 3.84	6.95 ± 3.70
5,12-DiHETE	AA	n-6	23.89 ± 104.27	17.91 ± 69.46
5-HETE	AA	n-6	217.81 ± 238.60	237.75 ± 358.43
5,15-DiHETE	AA	n-6	5.56 ± 13.25	25.34 ± 118.62
6-keto-PGF1a	AA	n-6	188.74 ± 576.20	317.97 ± 777.40
6-trans-LTB4	AA	n-6	17.09 ± 69.97	5.70 ± 18.53
6S-LXA4	AA	n-6	3.75 ± 6.13	4.71 ± 6.48
8-iso-PGF2a	AA	n-6	12.07 ± 14.92	11.78 ± 16.59
AEA	AA	n-6	677.98 ± 331.05	586.39 ± 388.39
LTB4	AA	n-6	14.16 ± 42.41	13.07 ± 36.70
LTE4	AA	n-6	18.89 ± 36.22	14.89 ± 31.60
LXB4	AA	n-6	15.35 ± 32.83	12.30 ± 23.96
PGD2	AA	n-6	61.07 ± 59.07	39.50 ± 78.93
PGE2	AA	n-6	139.76 ± 174.34	56.21 ± 73.36
PGF2a	AA	n-6	24.39 ± 19.44	17.43 ± 20.67
TXB2	AA	n-6	3982.52 ± 3414.11	2726.36 ± 5644.38
13-HOTrE	aLA	n-3	1948.25 ± 4569.58	1869.40 ± 4858.83
9-HOTrE	aLA	n-3	918.81 ± 2336.08	893.47 ± 2827.73
12-HETrE	DGLA	n-6	293.89 ± 557.74	192.19 ± 372.97
14,15-EpEDE	DGLA	n-6	30.67 ± 102.94	10.88 ± 23.46
15-HETrE	DGLA	n-6	37.74 ± 80.21	30.30 ± 92.73
8-HETrE	DGLA	n-6	639.25 ± 1500.77	345.19 ± 700.65
14-HDHA	DHA	n-3	1675.36 ± 2651.40	1389.05 ± 2570.04
16,17-EpDPA	DHA	n-3	47.47 ± 238.53	55.62 ± 177.77
17-HDHA	DHA	n-3	175.58 ± 791.38	106.18 ± 261.54
17R-RvD1	DHA	n-3	4.32 ± 6.96	5.04 ± 7.75
19,20-EpDPA	DHA	n-3	28.62 ± 64.79	26.88 ± 66.52
DHEA	DHA	n-3	641.83 ± 317.32	499.00 ± 298.11
Maresin1	DHA	n-3	233.71 ± 1225.39	72.56 ± 255.54
Maresin2	DHA	n-3	12.62 ± 35.66	51.87 ± 157.15
PD1	DHA	n-3	42.39 ± 98.81	35.76 ± 55.95
PDX	DHA	n-3	27.11 ± 89.47	12.50 ± 32.68
RvD1	DHA	n-3	6.17 ± 6.15	6.15 ± 7.13
RvD2	DHA	n-3	26.48 ± 39.82	25.64 ± 27.46
RvD3	DHA	n-3	8.85 ± 16.74	7.90 ± 11.44
RvD4	DHA	n-3	27.11 ± 66.63	31.65 ± 92.79
RvD5	DHA	n-3	58.38 ± 189.85	28.19 ± 70.57
17-oxo-DPA	DPA	n-3	1772.29 ± 4196.07	1840.87 ± 3129.45
n3 DPA RvD5	DPA	n-3	59.71 ± 124.71	41.46 ± 136.87
12-HEPE	EPA	n-3	307.48 ± 392.49	323.16 ± 667.17
15-HEPE	EPA	n-3	49.18 ± 77.28	66.09 ± 137.98
17,18-EpETE	EPA	n-3	202.18 ± 494.28	180.37 ± 468.81
18-HEPE	EPA	n-3	93.91 ± 210.78	126.86 ± 251.02
5-HEPE	EPA	n-3	268.68 ± 435.53	225.04 ± 290.66

EPEA	EPA	n-3	138.62 ± 91.93	119.89 ± 75.86
LTB5	EPA	n-3	27.57 ± 66.21	26.58 ± 60.19
PGD3	EPA	n-3	17.18 ± 51.96	25.91 ± 48.63
PGE3	EPA	n-3	52.69 ± 189.55	77.60 ± 382.41
RVE1	EPA	n-3	15.79 ± 11.98	16.44 ± 25.90
TxB3	EPA	n-3	80.30 ± 73.76	55.59 ± 99.73
12,13-EpOME	LA	n-6	13740.94 ± 16729.83	10477.51 ± 9800.64
13-HODE	LA	n-6	17431.60 ± 26538.97	17862.46 ± 17503.17
9-HODE	LA	n-6	18868.36 ± 35006.14	17216.12 ± 18811.58
9,10-EpOME	LA	n-6	680.34 ± 1405.53	520.86 ± 789.76

PUFA; Polyunsaturated fatty acid from which the corresponding oxylipin and endocannabinoid is derived from.

References

1. Schebb, N.H.; Kampschulte, N.; Hagn, G.; Plitzko, K.; Meckelmann, S.W.; Ghosh, S.; Joshi, R.; Kuligowski, J.; Vuckovic, D.; Botana, M.T., et al. Technical recommendations for analyzing oxylipins by liquid chromatography-mass spectrometry. *Sci Signal* **2025**, *18*, eadw1245, doi:10.1126/scisignal.adw1245.
